# Editorialets

**Published:** 1908-12

**Authors:** 


### Editorialets.

The Resignation of Dr. B. M. Worsham, as Superintendent of the State Lunatic Asylum, was a great surprise and is much to be regretted. It is a distinct loss to the State, for during his eighteen years' service—first at Austin as assistant physician, then as Superintendent at San Antonio, and for the past ten or more years at Austin—he has made himself a name as an asylum superintendent second to none in America. His administration has been successful, and he leaves that great institution in applepie order and working smoothly.

Dr. Worsham will settle in Austin, and study a year or so in Europe later.

The Tuberculosis Exhibit now in Texas will be exhibited in the principal cities of the State,—Austin, December 9th,—under the management of Dr. M. M. Carrick, of Dallas, an officer of the State Health Department. It is an object lesson that can not fail to arouse public interest in the great reform movement for the suppression of consumption. It is educational. The Legislature of Texas will doubtless be moved to do something to help the League in its noble fight.

For Sale.—Van Houten & Tenbroeck X-ray machine in good condition. Also one Betts' body hot-air machine, almost as good as new. These instruments will be sold at a great sacrifice. Apply to Texas Medical Journal, Austin, Texas.

Animal Therapy.—The *Bulletin-Journal of Animal Therapy* for November contains authentic reports of a large number of cases successfully treated by this method. The reports cover a wide range of diseases and are by men of reputation and known ability, many from Texas. They embrace pulmonary tuberculosis, bronchial asthma, locomotor ataxia, diabetes mellitus, sexual impotence, morphine addiction, neurasthenia and a score of other intractable diseases. Dr. Hawley, the editor, 72 Madison St., Chicago, will be pleased to send a copy of these reports (Bulletin) free to any physician on request. I append a few choice paragraphs from the bright little *Bulletin*:

"In top-rank medical journal contributions, non-surgical, the great guiding word is, be abstract, theorize, crowd the bibliography; *therapeutic contributions received at writer's risk.*

"By the way, you chemical counselors who are telling us the drugs to be chewed and those to be eschewed, can't you do something constructive, or suggest a remedy for present therapeutic coma?

"Honestly, doctor, don't you wish that these high-up journals would publish something more about how to help the sick, and less about 'A Study of Three Thousand Cases of Atypical Bibliography'?

"Oh, for a medical Moses to lead us out of the wilderness of words into the land of deeds! The medical journals publish annually a verbose mass of abstract theory and hypothetical rot sufficient to confuse a medical giant—their original therapeutic contributions wouldn't confuse a medical gnat.

"There must soon be a renaissance of therapeutic enthusiasm and fairness. It is sure to come eventually, but for every year it is delayed many more years will be required to overcome the inroads on legitimate medicine and human credulity which have been and are to be made by those who, knowing nothing about disease, treat it with illogical verbiage based upon a sacrilege of the scriptures, and those who, knowing a little about disease, treat it with blatant sophistry based upon a sacrilege of sense.

"Grand, old-time, general practitioner, you never had more than echo of adequate monetary compensation—your pay was largely genuine gratitude, respect, love. Overfilling of the profession, excessive specialism, and latter-day commercialism have greatly thinned your ranks, but you have left a great precept in the highest concept of medical practice. You have stood for the best in executive medicine and the best in potential citizenship. Your work was the concrete, you had *to do,* and in spite of inadequate, crude and largely empiric equipment, your results compare most favorably with those of today, and infinitely surpass those of the eminent agnostics who try to teach us treatment via the Great Abstract."

---

THE MANN BILL.—Look out for it and write your Senators and Congressmen to oppose it. It shows the "fine Italian hand" of the Octopus gang, the Bureaucrats in control. It prohibits the *sale to physicians* by manufacturers of hyoscine, duboisine, cocaine, scopalomine and all other "ines," the intent being to *compel doctors to prescribe* these things, instead of dispensing them. It is iniquitous and seems to be aimed especially at the manufacturers of alkaloids.

---

PELLAGRA, a disease caused by fungus or smut on corn. It is comparatively new and unknown to this country. The Public Health and Marine Hospital Service Department of the United States has issued a pamphlet, written by Dr. C. H. Lavender, Past Assistant Surgeon, at request of Surgeon General Wyman, covering the subject, and Dr. Wyman will send upon request, free of charge, a copy of it. It will interest you.

---

MR. EDITOR: Can you tell me why it is that we have a big Briton as third assistant editor of the *Journal of the American Medical Association?* Are there not enough Americans to edit the *Journal of the American Medical Association?* Are not the Americans the greatest journalists in the world, both medical and lay? Are there not more medical journals in America than all the rest of the world? I "dare say" and "I fancy" that it would be a "proper" cold day when an American is on the editorial staff of the *British Medical Journal.*

Respectfully,

E. S. MCKEE, M. D.,

Search *me.* No. I was never good at conundrums. I can't even tell you why an English-Irish Homeopath should be the *boss* editor of the *Jamie* (J. A. M. A.), nor why ye "American editors" who, you say, are "the greatest journalists in the world" should be afraid of him. I give it up.—EDITOR.

---

THE twenty-second annual session of the New Orleans Polyclinic opened on November 2nd with a larger class than ever before, and the indications are for a very successful term.

Dear Doctor Daniel: Enclosed check for $3, covering account and for one more year. Excuse my negligence, for I am with you in the fight against medical trusts. You, the *Medical World* and *Southern Clinic,* of Richmond, Va., are making converts every month. Yours fraternally,

J. R. Evans, Devine, Texas.

---

Menorrhagia.—The desideratum for the relief of this condition is a remedy which will not only stimulate contraction, but will impart tone to the uterus as well. Such a remedy is Hayden's Viburnum Compound. Its action is superior to and far more lasting than ergot and is devoid of the toxic effects of this drug.

